# A prospective, multi-site, observational study assessing the treatment thresholds of new onset atrial fibrillation in the critically ill

**DOI:** 10.1016/j.ccrj.2026.100188

**Published:** 2026-05-22

**Authors:** H.G.M. Walker, N.P. Anthony, J. Reeve, C. McDermott, M. Fogarty, P. Emerson, J. Walker, T. Evans, J. Holmes, K.J. Denny, A. Brown, the OPEN-ICU Network

**Affiliations:** a Department of Critical Care Medicine, St Vincent's Hospital, Melbourne, Victoria, Australia; b Department of Critical Care, University of Melbourne, Victoria, Australia; c Intensive Care Unit, Epworth Richmond, Victoria, Australia; d Intensive Care Unit, Fiona Stanley Hospital, Western Australia, Australia; e Intensive Care Unit, Austin Hospital, Victoria, Australia; f Intensive Care Unit, Bendigo Health, Victoria, Australia; g Department of Intensive Care Medicine, Royal Adelaide Hospital, South Australia, Australia; h Intensive Care Unit, Gold Coast University Hospital, Queensland, Australia; i Intensive Care Unit, Royal Melbourne Hospital, Victoria, Australia; j School of Medicine, University of Western Australia, Western Australia, Australia; k School of Public Health and Preventive Medicine, Monash University, Victoria, Australia

**Keywords:** Atrial fibrillation, New-onset atrial fibrillation, Critical illness, Antiarrhythmics, Rate control

## Abstract

**Objective:**

To define haemodynamic thresholds for pharmacological treatment of new-onset atrial fibrillation (NOAF) in critically ill patients and examine associations with short-term outcomes including reversion to sinus rhythm and in-hospital mortality.

**Design, setting, and participants:**

prospective multicentre observational cohort study conducted across six Australian intensive care units (ICUs) from December 2024 to September 2025. Adult patients (≥18 years) who developed NOAF during ICU admission were included. A Cox proportional hazards model treating time to treatment as a time-dependent covariate was used to assess associations between pharmacological therapy and secondary outcomes.

**Main outcome measures:**

The primary outcome was heart rate at initiation of the first pharmacological therapy for NOAF. Secondary outcomes included mean arterial pressure at time of treatment, reversion to sinus rhythm, haemodynamic deterioration, ICU length of stay, and in-hospital mortality.

**Results:**

Four hundred eighty-two patients were screened for eligibility and 210 met the inclusion criteria. One hundred fifty-four (73.3%) received pharmacological treatment for NOAF. The median heart rate at treatment initiation was 128 beats per minute (interquartile range [IQR]: 113-147) and median mean arterial pressure 76 mmHg (IQR: 70-87). Pharmacological treatment was not independently associated with sustained reversion to sinus rhythm (adjusted hazard ratio: 1.44, 95% confidence interval [CI]: 0.94-2.20) or in-hospital mortality (adjusted odds ratio: 0.96, 95% CI: 0.36-2.58). Pharmacological treatment was associated with prolonged ICU stay (multiplicative adjusted effect: 1.39, 95% CI: 1.06-1.82).

**Conclusions:**

Pharmacological treatment for NOAF is typically initiated at heart rates approximating 130 bpm. After accounting for time-dependent exposure, pharmacological treatment was not independently associated with reversion to sustained sinus rhythm. Given the methodological limitations encountered, these findings warrant validation in randomised trials to better define optimal treatment strategies for NOAF in critical illness.

## 1 Introduction

New-onset atrial fibrillation (NOAF) is the occurrence of atrial fibrillation (AF) in patients without a prior diagnosis of AF.^1^ It is common among patients who are critically ill in the intensive care unit (ICU), with reported incidences ranging from 5 to 46%.^2^ Most recently, a large observational study of nearly 47,000 patients in Queensland, Australia, reported an incidence of 8.4%.^3^ Patients who experience NOAF are at increased risk of mortality[4], [5], [6] and other adverse events such as thromboembolism^7^ or subsequent hospitalisation with AF, heart failure, and stroke.^5^ Several authors have identified the need for higher quality data with respect to incidence, outcomes, and management of NOAF in the critically ill.^2^^,^^8^^,^^9^ Multiple international surveys have demonstrated that critical care clinicians report different heart rate thresholds for intervention and timing of the initiation of anti-arrhythmic drugs^10^^,^^11^; however, these thresholds have not been assessed in dedicated studies of clinical practice. There is also substantial variation in the choice of pharmacological agents used in the management of NOAF in the critically ill. AFIB-ICU^12^ was a large cohort study that assessed NOAF in the critically ill. However, it only included one centre in Australia and New Zealand and did not capture vital signs, such as heart rate and blood pressure, at the time of the initiation of therapy. More recently, an observational study of 33 French ICUs found that whilst treatment was mostly initiated within 30 min,^13^ the actual haemodynamic thresholds at the time of treatment were unknown. Uncertainty around optimal timing and nature of therapy for NOAF has recently been highlighted as an important knowledge gap and research opportunity.^14^ Our objective was to clarify thresholds around initiation of pharmacological therapy in critical illness–associated NOAF in Australia and examine associations with short-term outcomes such as reversion to sinus rhythm, length of stay, and in-hospital mortality.

## 2 Methods

### 2.1 Study design and setting

This was a prospective observational cohort study conducted in six Australian ICUs between December 2024 and September 2025. Five of these were tertiary ICUs and admitted cardiac surgical patients; one was a regional centre without cardiac surgery. Ethical approval was granted by St Vincent’s Hospital, Melbourne Human Research and Ethics Committee (2024/PID00172, 16th July 2024). A waiver of informed consent was granted as all data were routinely collected clinical variables. The OPEN-ICU network is a network aimed at providing opportunities for novice research clinicians to undertake observational research supervised by more experienced clinicians.^15^

### 2.2 Study population

All adult ICU patients (≥18 years) who developed AF with rapid ventricular rate (RVR) greater than 100 beats per minute (bpm) during ICU admission were screened and eligible for inclusion. Patients were excluded if they had a prior history of AF, received dedicated pharmacological treatment before ICU admission, the episode occurred on a readmission to ICU, they were admitted with palliative intent, or were transferred from a nonparticipating ICU.

### 2.3 Outcomes

The primary outcome was heart rate at initiation of first pharmacological treatment for NOAF. Secondary outcomes included time to treatment, haemodynamic deterioration within 6 h of NOAF onset, sustained reversion to sinus rhythm, ICU and hospital length of stay (LOS), and in-hospital mortality.

### 2.4 Sample size

No formal sample size calculation was conducted and convenience sampling was used. Based on reported incidence of ten episodes per month at our pilot site (St Vincent’s Hospital, Melbourne), we initially planned to recruit for 3 months at eight sites. However, due to logistical issues, recruitment was slower than anticipated at only six sites, so the recruitment period was extended up to six months.

### 2.5 Definitions and identification

NOAF was defined as an irregular rhythm with an RVR (>100 bpm), an absence of p waves and irregular R–R intervals that was identified by continuous monitoring or 12-lead electrocardiogram (ECG) lasting at least 30 s.^16^ To ensure no cases were missed by local investigators, data from the electronic medical record were also extracted to identify any additional patients in which bedside nurses recorded AF as a rhythm. This has been shown to be an accurate method of identifying patients in AF.^17^ These additional patients were then reviewed by investigators at each site to ensure they were eligible. Pharmacological treatment was limited to specific medications for rate and/or rhythm control (i.e. electrolyte replacement was not defined as receiving pharmacological therapy). Sustained reversion was defined as a period of at least 24 h without AF^18^ (or discharged from ICU in sinus rhythm if this occurred before 24 h). Haemodynamic deterioration was defined as a composite of either a new systolic blood pressire (SBP) <90 mmHg, a new requirement for vasopressors, an increase in vasopressor requirements (>5 mcg/min of noradrenaline or 3 mg/h of metaraminol based on a 10:1 conversion^19^), or the addition of a second vasopressor. This had to be clinically perceived by the local investigator as related to the arrhythmia. This was felt to be a pragmatic definition given the limited literature describing NOAF-related haemodynamic instability.^20^ All other definitions are provided in the supplementary appendix.

### 2.6 Statistical analysis

A statistical analysis plan was finalised prior to database locking and analysis. All analyses were conducted using R Version 4.5.1 (R Core Team, Vienna, Austria). Continuous variables were summarised as mean (standard deviation) or median (interquartile range [IQR]) and categorical variables as frequency (%). Between-group comparisons used Wilcoxon rank-sum tests for non-normally distributed variables and chi-squared or Fisher's exact tests for categorical variables. Vasopressor dose was converted to noradrenaline equivalents using a recognised formula.^21^ Time-to-event outcomes were analysed using Cox proportional hazards models; pharmacological treatment was modelled as time-dependent (0 prior to treatment, 1 after) to reduce immortal time bias^22^ as patients who are sicker may receive pharmacological treatment earlier. The proportional hazards assumption was assessed using Schoenfeld residuals. In order to visualise these relationships, unadjusted survival curves were used. ICU LOS was analysed using a log-linear model; exponentiated coefficients were interpreted as multiplicative effects on LOS. In-hospital mortality was analysed using mixed-effects logistic regression with a random intercept for site. Covariates were determined *a priori* and based on clinical relevance and available evidence. These included sex, Acute Physiology, Age and Chronic Health Evaluation (APACHE) III score, recent cardiac surgery, and vasopressor use at the time of NOAF onset. Discharge from ICU in NOAF was included as an additional covariate in the model assessing associations with in-hospital mortality. Site-level clustering was summarised using the intraclass correlation coefficient computed via the latent variable method (residual variance: π^2^/3). For the primary outcome, five prespecified subgroups (presence or absence of vasopressors, a history of recent cardiac surgery, presence of a central venous catheter, age, and the use of an antiarrhythmic prior to ICU admission that is used for an indication other than AF) were analysed to see if there was a difference in the heart rate at treatment initiation. A p value of <0.05 was considered significant.

## 3 Results

### 3.1 Study population

**Fig. 1:**
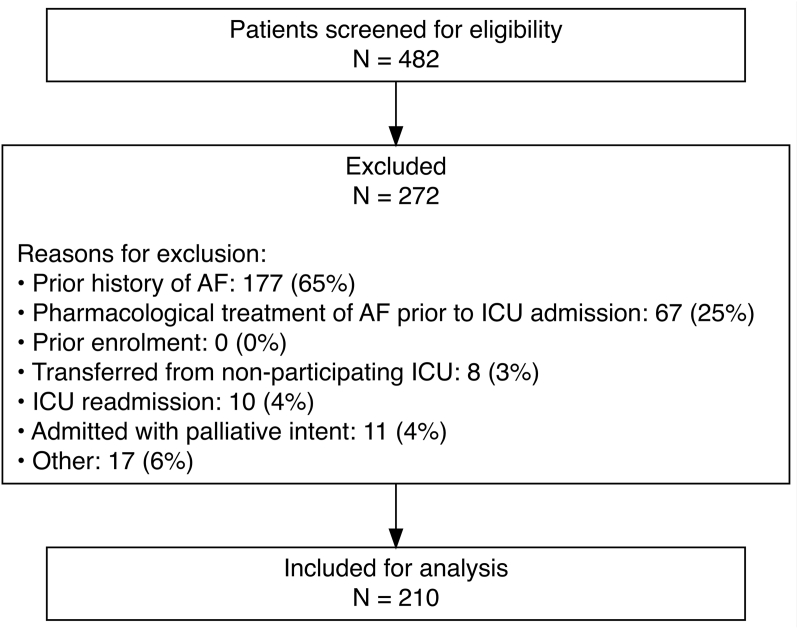
Figure showing participant flow through the study. Numbers and percentages do not add up to 272 or 100% as categories were not exclusive. “Other” includes HR < 100bpm (n = 10), unable to consent (n = 4), in ICU prior to study commencement (n = 2), and not AF (n = 1). HR – Hazard Ratio; ICU – Intensive Care Unit.

Of 482 ICU patients who developed AF with RVR during the study period, 272 (56.4%) were excluded, primarily due to the pre-existing AF (177, 65%), leaving 210 patients for analysis (Fig. 1). Of these, 154 (73.3%) received pharmacological treatment for NOAF and 56 (26.6%) did not. Baseline demographics are shown in Table 1. For the overall population, the mean age was 65.3 (standard deviation: 12.2) years and 31.4% were female. With respect to admission source, 109 (51.9%) were admitted from the operating theatre and 67 (31.9%) had undergone cardiac surgery. At the time of NOAF onset, 63 (30.0%) were invasively ventilated and 77 (37.0%) were receiving vasopressors. Between the treated and non-treated groups, baseline characteristics were broadly similar except a history of ischaemic heart disease was more common in the treated group (31.8% vs 17.9%, *p* = 0.047). Co-interventions are reported in Table S1, a greater proportion in those who did not receive pharmacological treatment received IV electrolytes (55 vs 71%, p = 0.034).

**Table 1:** Baseline demographics stratified by whether pharmacological therapy for NOAF was received.

	Overall N = 210	No pharmacological treatment N = 56	Pharmacological treatment N = 154	p value
**Age, years**	65.3 (12.2)	65.5 (13.8)	65.2 (11.6)	0.6
**Female sex, n (%)**	66 (31.4%)	13 (23.2%)	53 (34.4%)	0.12
**Admission source, n (%)**				0.061
ED	54 (25.7%)	22 (39.3%)	3 (20.8%)	
Ward	37 (17.6%)	9 (16.1%)	28 (18.2%)	
OT	109 (51.9%)	23 (41.1%)	86 (55.8%)	
Other	10 (4.8%)	2 (3.6%)	8 (5.2%)	
**Admission type, n (%)**				0.2
Medical	103 (49.8%)	33 (61.1%)	70 (45.8%)	
Non-elective surgery	35 (16.9%)	7 (13.0%)	28 (18.3%)	
Elective surgery	69 (33.3%)	14 (25.9%)	55 (35.9%)	
**Mean APACHEIII score (SD)**	55.0 (27.4)	56.4 (29.2)	54.5 (26.8)	0.6
**Comorbidities, n (%)**				
Hypertension	121 (57.6%)	27 (48.2%)	94 (61.0%)	0.11
Ischaemic heart disease	59 (28.1%)	10 (17.9%)	49 (31.8%)	0.056
Recent cardiac surgery	67 (31.9%)	12 (21.4%)	55 (35.7%)	0.065
Tachyarrhythmia (not AF)	4 (1.9%)	3 (5.4%)	1 (0.6%)	0.059
Cardiac device^a^	4 (1.9%)	2 (3.6%)	2 (1.3%)	0.3
Chronic liver disease	4 (1.9%)	1 (1.8%)	3 (1.9%)	>0.9
Malignancy	30 (14.3%)	7 (12.5%)	23 (14.9%)	0.8
Prior VTE	8 (3.8%)	2 (3.6%)	6 (3.9%)	>0.9
Prior use of anti-arrhythmics, n (%)^b^	56 (26.7%)	15 (26.8%)	41 (26.6%)	>0.9
**Invasively ventilated at time of NOAF onset, n (%)**	63 (30.0%)	21 (37.5%)	42 (27.3%)	0.2
**CVC in situ at time of NOAF onset, n (%)**	161 (77.4%)	37 (66.1%)	124 (81.6%)	0.024
**Receiving vasopressors at time of NOAF onset, n (%)**	77 (37.0%)	25 (44.6%)	52 (34.2%)	0.2
**Mean potassium level prior (mmol/L), mean (SD)**	4.23 (0.60)	4.21 (0.64)	4.24 (0.59)	0.4
**Magnesium level prior (mmol/L), mean (SD)**	1.03 (0.27)	1.02 (0.31)	1.03 (0.25)	0.5

APACHE - Acute Physiology, Age and Chronic Health Evaluation; ED – Emergency Department; OT – Operating Theatre; VTE – Venous thromboembolism; NOAF – New Onset Atrial Fibrillation, CVC – Central Venous Catheter; SD – Standard Deviation.

a Includes a permanent pacemaker or internal cardiac defibrillator.

b For uses other than atrial fibrillation (AF).

### 3.2 Primary outcome

Among treated patients (n = 154), the median heart rate at initiation of pharmacological therapy was 128 (IQR: 113-147) bpm.

### 3.3 Other haemodynamic measurements and treatment thresholds

**Fig. 2:**
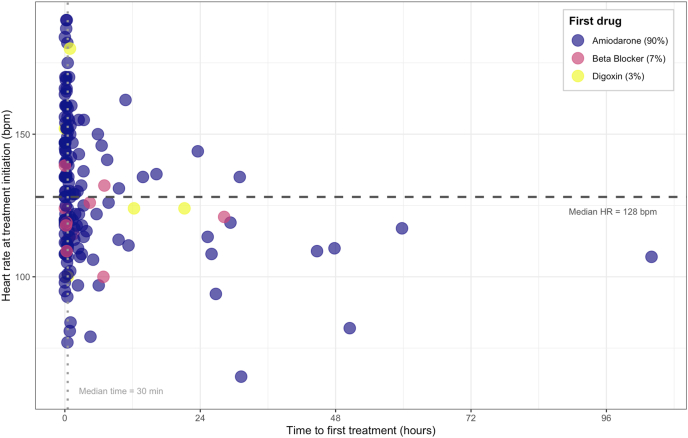
showing time to initiation of first pharmacological therapy (x-axis), heart rate at initiation of therapy (y-axis), and first drug used (colour).

The median mean arterial pressure was 76 mmHg (IQR: 70-87) (Fig. 2, Table 2). The median time to treatment was 30 (IQR: 10-180) minutes. Vasopressor dose at NOAF onset was higher among treated patients (noradrenaline equivalents of 10.0 (4.0–21.8) vs 5.0 (3.0–9.0) mcg/min, p = 0.037). The treated patients had higher maximal heart rates within 24 h (140 vs 116 bpm, p < 0.001).Heart rate at treatment initiation was broadly similar across prespecified subgroups (Figure S1). Of the 154 patients treated, amiodarone was the most frequently used first-line agent in 138 patients (90%), followed by beta-blockers (n = 11, 7%) and digoxin (n = 5, 3%). Only 32 (20.8%) of those treated received a second antiarrhythmic during their stay with a median time of 9.7 [3.7-29.8] hours from administration of their first drug (Figure S2).

**Table 2:** NOAF treatments and outcomes stratified by whether pharmacological therapy received.

	No pharmacological treatment N = 56	Pharmacological Treatment N = 154	p value
HR at time of treatment, bpm	–	128.0 (113.0–147.0)	
MAP at time of treatment, mmHg	–	76.0 (70.0–87.0)	
Vasopressor dose at NOAF onset, noradrenaline equivalents (mcg/min) a	5.0 (3.0–9.0)	10.0 (4.0–21.8)	0.037
Time to treatment, hours	–	0.5 (0.2–3.0)	
Maximal HR in first 24 h post onset	116.0 (101.0–136.0)	140.0 (122.5–154.0)	<0.001
Haemodynamic deterioration ≤6h after NOAF onset, n (%)	5.0 (8.9%)	31.0 (20.1%)	0.057
Any Documented reversion to SR, n (%)	43 (76.8%)	128 (83.7%)	0.3
Sustained reversion to SR, n (%)	34 (79.1%)	81 (63.8%)	0.064
Time to sustained SR, hours	3.0 (2.0–13.0)	6.0 (3.0–10.0)	0.12
Discharged ICU in AF, n (%)	12.0 (21.4%)	36.0 (23.8%)	0.7
ICU LOS (days)	4.1 (2.3–6.5)	4.8 (2.9–8.7)	0.10
Hospital LOS (days)	14.2 (7.3–28.1)	13.7 (8.3–27.1)	0.9
Hospital mortality, n (%)	9.0 (17.6%)	23.0 (15.8%)	0.8

All are median (IQR) unless specified.

bpm – Beats per Minute; mcg/min – Micrograms per Minute; HR – Heart Rate; MAP – Mean Arterial Pressure; SR – Sinus Rhythm; ICU – Intensive Care Unit; LOS – Length of Stay.

a To calculate noradrenaline equivalents, the equation by Goradia et al.^21^ was used; however, to ensure consistency with the definition for haemodynamic deterioration and for metaraminol. a conversion ratio of 10:1^19^ was used.

### 3.4 Secondary outcomes

Overall rates of any reversion to sinus rhythm were similar between treated and untreated patients (83.7% vs 76.8%, p = 0.3, Table 2). In those treated, the median time to reversion was 6 (3–10) hours, compared to 3 (2–13) hours in those not treated (p = 0.12) (Figure S3). Pharmacological treatment was not independently associated with sustained reversion (adjusted hazard ratio, aHR: 1.44, 95% confidence interval [CI]: 0.94-2.20). No violation of the proportional hazards assumption was demonstrated (global *p* = 0.25, Table S2). Vasopressor use at onset was associated with a significantly lower probability of sustained reversion (aHR: 0.59, 95% CI: 0.39-0.91) (Table 3).

**Table 3:** Cox proportional hazard model showing the association of pharmacological treatment with sustained reversion to sinus rhythm.

Characteristic	Unadjusted HR	95% CI	Adjusted HR	95% CI
Pharmacological treatment (time-dependent)	1.28	0.85, 1.92	1.44	0.94, 2.20
Sex (male)	1.18	0.79, 1.77	1.40	0.92, 2.14
APACHE III	1.00	0.99, 1.01	1.00	0.99, 1.01
Recent cardiac surgery	0.91	0.60, 1.38	0.89	0.57, 1.38
Vasopressor use	0.65	0.43, 0.98	0.59	0.39, 0.91

CI– Confidence Interval, HR– Hazard Ratio, APACHE– Acute Physiology, Age, and Chronic Health Evaluation.

Pharmacological therapy was associated with a significantly longer ICU stay (multiplicative adjusted effect: 1.39, 95% CI: 1.06-1.82), which corresponds to an approximate 39% increase in the ICU length of stay compared with patients who did not receive pharmacological treatment (Table S3). In a mixed-effects logistic regression model with a random intercept for site that was adjusted for gender, illness severity (APACHE III score), a prior history of cardiac surgery, and vasopressor use, treatment was not associated with in-hospital mortality (adjusted OR: 0.96, 95% CI: 0.36-2.58) (Table S4 and S5). When considering haemodynamic deterioration within 6 h of NOAF onset, the rates of haemodynamic deterioration were 20.1% in those who received pharmacological treatment, compared to 8.9% in those not administered pharmacological therapy (p = 0.057). Of those that did receive pharmacological treatment, haemodynamic deterioration occurred after drug administration in 22 (71.0%) patients.

## 4 Discussion

### 4.1 Principal findings

In this prospective multicentre cohort of Australian ICU patients, the administration of pharmacological treatment for NOAF was common and typically initiated early and at a median heart rate of 128 bpm. High rates of spontaneous reversion were demonstrated in both groups. Patients who received pharmacological therapy exhibited greater physiological acuity, including higher maximal heart rate, greater vasopressor exposure, and higher rates of haemodynamic deterioration. After accounting for time-dependent exposure, pharmacological treatment was not independently associated with sustained reversion to sinus rhythm or in-hospital mortality. Vasopressor use at the time of NOAF onset was consistently associated with reduced rates of sustained reversion, longer ICU LOS, and increased mortality, underscoring the role of shock physiology in shaping NOAF trajectories and outcomes.

### 4.2 Relationship to prior literature

The heart rate treatment thresholds in this study are consistent with the previous thresholds reported in international clinician surveys but extend this literature by demonstrating how these thresholds are applied in clinical practice. In the survey by Wetterslev et al.,^11^ 45% of respondents indicated that they would initiate treatment at heart rates between 110 and 129bpm; while Johnston et al.^10^ reported that 48% of respondents would initiate treatment at heart rates between 120 and 139 bpm. Neither survey addressed blood pressure treatment thresholds. Similarly, consistent with a recent study by Pardo et al.,^13^ NOAF was treated *early* following onset of the arrhythmia, with a median time to treatment of 30 min.

Amiodarone was overwhelmingly the first-line agent in this cohort, mirroring both survey data and prior observational studies.^3^ This likely reflects clinician comfort and familiarity with amiodarone across ICU settings. Nonetheless, the lack of adequately powered randomised controlled trials comparing first-line pharmacological agents for NOAF in the setting of critical illness limits the ability to provide recommendations as to optimal therapy.^20^

While amiodarone has a slower onset of action than beta-blockers,^2^ prior observational data suggest similar overall efficacy for rate or rhythm control, and superiority compared to digoxin and calcium channel blockers.^23^ Importantly, concerns that beta-blockers are more likely to cause hypotension have not been consistently supported in critically ill populations,^24^ including patients with sepsis-associated AF.^25^ This may explain the low rates of beta-blocker use within our study.

In our cohort, pharmacological treatment was not independently associated with sustained reversion to sinus rhythm, whereas vasopressor use at NOAF onset reduced the likelihood of sustained reversion to sinus rhythm. This pattern is biologically plausible. Catecholamine exposure, systemic inflammation, metabolic derangements, atrial stretch, and organ dysfunction, all of which are common in patients with shock, are potent arrhythmogenic drivers.^2^^,^^9^^,^^14^^,^^26^ In this context, NOAF may represent a manifestation of underlying physiological instability rather than a discrete, readily modifiable arrhythmia.^27^ This supports the hypothesis that NOAF in critical illness is often an epiphenomenon, rather than a primary therapeutic target.^27^

### 4.3 Interpretation of haemodynamic deterioration and treatment effects

Whilst the rates of haemodynamic deterioration were not statistically different, in approximately two-thirds of those receiving pharmacological treatment, haemodynamic deterioration occurred following drug administration. While this temporary relationship may appear to be a concerning adverse effect of pharmacological therapy, it may also be explained by confounding by indication given the higher maximum heart rates in the 24 h following NOAF onset and higher vasopressor doses at baseline. Clinicians are likely to initiate treatment in patients showing worsening physiological parameters or perceived risk of instability. As such, pharmacological therapy may serve as a marker of clinical deterioration rather than its cause.

The lack of any observed association between treatment and improved outcomes (reversion or mortality) need to be interpreted with caution given the likely confounding by indication. Whilst no inference regarding the net benefit of early pharmacological intervention can be concluded, these findings highlight the potential importance of addressing underlying drivers such as shock and inflammation in determining outcomes such as reversion to sinus rhythm, regardless of therapies used.

### 4.4 Implications

Most prior studies have compared outcomes of patients with NOAF to those with no history of NOAF^28^^,^^29^ or pre-existing AF,^30^ rather than evaluating outcomes according to receipt of pharmacological therapy. This distinction is critical given the rapid initiation of treatment and the high rates of reversion (irrespective of therapy) demonstrated in this and prior studies.^31^ Commonly administered pharmacological treatments for NOAF are not necessarily benign in the critically ill. Amiodarone, the most commonly utilised pharmacological therapy for NOAF used in our cohort, carries recognised risks, including pulmonary toxicity^32^ and prolonged QT interval, which may occur early in therapy.^33^ Moreover, initiation of amiodarone in the ICU is frequently followed by prolonged continuation without specialist review or clear outpatient follow-up plans.^34^

Taken together, these data underscore the need for randomised clinical trials to define optimal management strategies for NOAF in critical illness. Future studies should explore early versus delayed pharmacological therapy for NOAF or alternative strategies that integrate haemodynamic status, vasopressor exposure, and arrhythmia burden to better identify patients most likely to benefit from pharmacological intervention.^35^ Advances in continuous monitoring modalities, such as wearable cardiac monitoring may facilitate more precise phenotyping of NOAF both during and after ICU admission.^36^ Given the recognised long-term sequelae^37^ of NOAF, including recurrent AF, stroke, and heart failure, trials that link ICU-based treatment strategies for NOAF with structured post-discharge follow-up are likely to have substantial implications for both patients and healthcare systems.

### 4.5 Strengths and limitations

This study has several important strengths. First, this study identifies haemodynamic thresholds for NOAF treatment that are used in contemporary clinical practice, rather than relying on clinician’s self-reporting thresholds. This provides critical information for the design of future interventional trials. Second, this was a multicentre study across four Australian states and included one regional site, enhancing the generalisability of these results. Third, missing data was minimal. Fourth, the use of time-dependent modelling reduced immortal time bias when assessing treatment effects. Finally, analyses were conducted according to a prespecified analysis plan finalised prior to the database being locked.

Several limitations merit consideration. First, the observational design precludes causal inference and leaves residual confounding by indication unavoidable. Differential use of pharmacological agents in those deemed at greater risk of deterioration or less likely to spontaneously revert cannot be fully addressed by the model. Equally, whilst baseline APACHE III scores were similar, greater vasopressor dosage at NOAF onset suggests a greater illness severity in the treatment group, which may also account for longer ICU length of stay. Therefore, without more robust adjustment methods, longer ICU stay should not be characterised as a treatment-related harm. Second, whilst we collected data on some predisposing conditions for NOAF, we were unable to collect data on all potential risk factors and therefore unmeasured confounding likely remains. Third, mortality and site-level analyses were limited due to modest event counts, and the predominance of amiodarone restricted comparison among pharmacological agents. Fourth, CIs were wide and include both potential benefit and harm. Fifth, no formal power calculation was conducted. Given these limitations, the secondary outcomes should only be considered hypothesis generating. Sixth, we were neither able to assess AF recurrence following ICU discharge nor clinician intent regarding rhythm or rate control. Seventh, the definition of haemodynamic deterioration, while pragmatic, may not capture transient or undocumented events and more complex treatment–deterioration relationships could not be modelled. Additionally, there was subjectivity in the definition. Eighth, whilst we tried to capture all cases of NOAF, it is possible that transient episodes may have been undetected by bedside staff. This is more likely in the untreated group, which may introduce an ascertainment bias. Finally, we did not capture any NOAF-related harms beyond haemodynamic deterioration, which is important given the evidence around longer-term morbidity (e.g. thromboembolism, subsequent hospitalisations) associated with both NOAF and its treatment.

## 5 Conclusion

Pharmacological treatment for NOAF in Australian ICUs is commonly initiated at heart rates approximating 130 bpm and at a median of 30 min from the onset. In those receiving pharmacological treatment, vasopressor dose was higher at NOAF onset. After accounting for time-dependent exposure, pharmacological treatment was not independently associated with reversion to sustained sinus rhythm. Given the methodological limitations encountered, these findings warrant validation in randomised trials that focus on clinically relevant endpoints to better define optimal treatment strategies for NOAF in critical illness.

## CRediT authorship contribution statement

***Humphrey Walker****:* Conceptualisation, Supervision, Project administration, Methodology, Investigation, Validation, Writing – original draft, Data collection.

***Tess Evans:*** Conceptualisation, Methodology, Writing – review & editing.

***Kerina Denny:*** Conceptualisation, Methodology, Writing – review & editing.

***Alastair Brown:*** Conceptualisation, Methodology, Writing – review & editing.

***Nicholas Anthony****:* Writing – original draft, Validation, Formal analysis, Data curation, Visualisations, Data collection.

***Jennifer Holmes:*** Project administration, Methodology, Writing – review and editing.

***Jake Reeve, Conor McDermott, Mark Fogarty, Phil Emerson:*** Data collection, Writing – review & editing.

***Jake, Conor, Mark and Phil:*** Data collection, Writing - review and editing.

## Funding

No funding was received for this project.

## Conflict of interest

The authors declare that they have no known competing financial interests or personal relationships that could have appeared to influence the work reported in this paper.
