# Supplementary material for: A prospective, multi-site, observational study assessing the treatment thresholds of new onset atrial fibrillation in the critically ill
Source: Crit Care Resusc. 2026 May 22;28(2):100188. doi: 10.1016/j.ccrj.2026.100188 (PMC13223826; doi:10.1016/j.ccrj.2026.100188)
Supplement: Multimedia component 1 [file mmc1.docx]

**A prospective, multi-site, observational study assessing the treatment thresholds of new onset atrial fibrillation in the critically ill**

Walker HGM ^1,2, 3^, Anthony NP ^4^, Reeve J ^1^, McDermott C ^5^, Fogarty M ^6^, Emerson P ^7^, Walker J ^8^, Evans T ^2,9,10^, Holmes J ^1^, Denny KJ ^8^ , Brown A ^1,2,5,11^ on behalf of OPEN-ICU Network

**Affiliations**

1. Department of Critical Care Medicine, St Vincent’s Hospital Melbourne, Victoria
2. Department of Critical Care, University of Melbourne, Victoria
3. Intensive Care Unit, Epworth Richmond, Victoria
4. Intensive Care Unit, Fiona Stanley Hospital, Western Australia
5. Intensive Care Unit, Austin Hospital, Victoria
6. Intensive Care Unit, Bendigo Health, Victoria
7. Department of Intensive Care Medicine, Royal Adelaide Hospital, South Australia
8. Intensive Care Unit, Gold Coast University Hospital, Queensland
9. Intensive Care Unit, Royal Melbourne Hospital, Victoria
10. School of Medicine, University of Western Australia, Western Australia
11. School of Public Health and Preventive Medicine, Monash University, Victoria,

**Table S1: Co-interventions**

| **Non-pharmacological interventions ^a^** | **Overall**  N = 210 | **No Pharmacological Treatment**  N = 154 | **Pharmacological Treatment** *^a^*  N = 56 | **p- value** |
| --- | --- | --- | --- | --- |
| IV Electrolyte Replacement, n (%) | 125 (60%) | 85 (55%) | 50 (71%) | 0.034 |
| Fluid Bolus, n (%) | 19 (9.0%) | 11 (7.1%) | 8 (14.0%) | 0.11 |
| Change in Antimicrobials, n (%) | 114 (55%) | 5 (3.2%) | 0 (0.0%) | 0.3 |
| DC Cardioversion, n (%) *^b^* | 7 (3.3%) | 7/153 (4.6%) | 0 (0.0%) | 0.2 |

IV = intravenous, DC = Direct Current

*^a^* With the exception of DC Cardioversion all of these were before any pharmacological therapy was administered

*^b^* DC cardioversion events only occurred after pharmacological treatment. 3 patients had one shock, 3 patients had 2 shocks, and 1 had three shocks. Energy used ranged from 20 – 200J. 3 patients with DCR had sustained reversion and 3 did not (data was missing for one patient).

**Table S2: Schoenfeld Residuals**

| **Variable** | **Chi-square** | **df** | **p-value** |
| --- | --- | --- | --- |
| Pharmacological treatment | 0.13 | 1 | 0.723 |
| Sex | 2.65 | 1 | 0.104 |
| APACHE III | 0.49 | 1 | 0.484 |
| Cardiac surgery | 0.57 | 1 | 0.452 |
| Vasopressor use | 1.15 | 1 | 0.284 |
| ***Global test*** | 6.63 | 5 | 0.250 |

**Table S3: Association between pharmacological treatment and ICU LOS**

| **Variable** | **Unadjusted Multiplicative Effect Size (95% CI)** | **Adjusted Multiplicative Effect Size (95% CI)** |
| --- | --- | --- |
| Pharmacological Rx | 1.24 (0.94 – 1.64) | 1.39 (1.06-1.82) |
| Sex: Male | 1.10 (0.85 – 1.44) | 1.08 (0.83-1.39) |
| APACHE III (per point) | 1.01 (1.00 – 1.01) | 1 (1-1.01) |
| Recent Cardiac Surgery: Yes | 0.78 (0.60 – 1.01) | 0.76 (0.59-0.99) |
| Vasopressors: Yes | 1.55 (1.21 – 1.99) | 1.62 (1.26-2.07) |

**Table S4. Risk-adjusted association with in-hospital mortality using mixed-effects logistic regression with random intercept for site**

| **Characteristic** | **Unadjusted OR (95% CI)** | **Adjusted OR (95% CI)** |
| --- | --- | --- |
| Pharmacological treatment | 0.83 (0.34 – 2.01) | 0.96 (0.36-2.58) |
| Male | 1.01 (0.44 – 2.30) | 0.73 (0.29-1.86) |
| APACHE III (per point) | 1.04 (1.02 – 1.06) | 1.03 (1.00-1.06) |
| Vasopressor use | 3.72 (1.65 – 8.39) | 4.33 (1.75-10.69) |
| Recent cardiac surgery | 0.33 (0.12 – 0.92) | 0.46 (0.14-1.45) |
| Discharged ICU in atrial fibrillation | 0.93 (0.37 – 2.36) | 1.05 (0.38-2.85) |

Adjusted odds ratios (OR) with 95% confidence intervals from a mixed-effects logistic regression model (random intercept for site).

**Table S5. Intraclass Correlation Coefficient (ICC) for In-Hospital Mortality**

| **Model** | **Site-level variance (σ^2^)** | **ICC** |
| --- | --- | --- |
| Null model (site only) | 0.068 | 0.020 |
| Risk-adjusted model | 0.790 | 0.194 |

ICC calculated using the latent variable method for logistic mixed-effects models, assuming residual variance π^2^ / 3

**Figure S1 showing median heart rate (and IQR) at treatment in pre-specified subgroups**


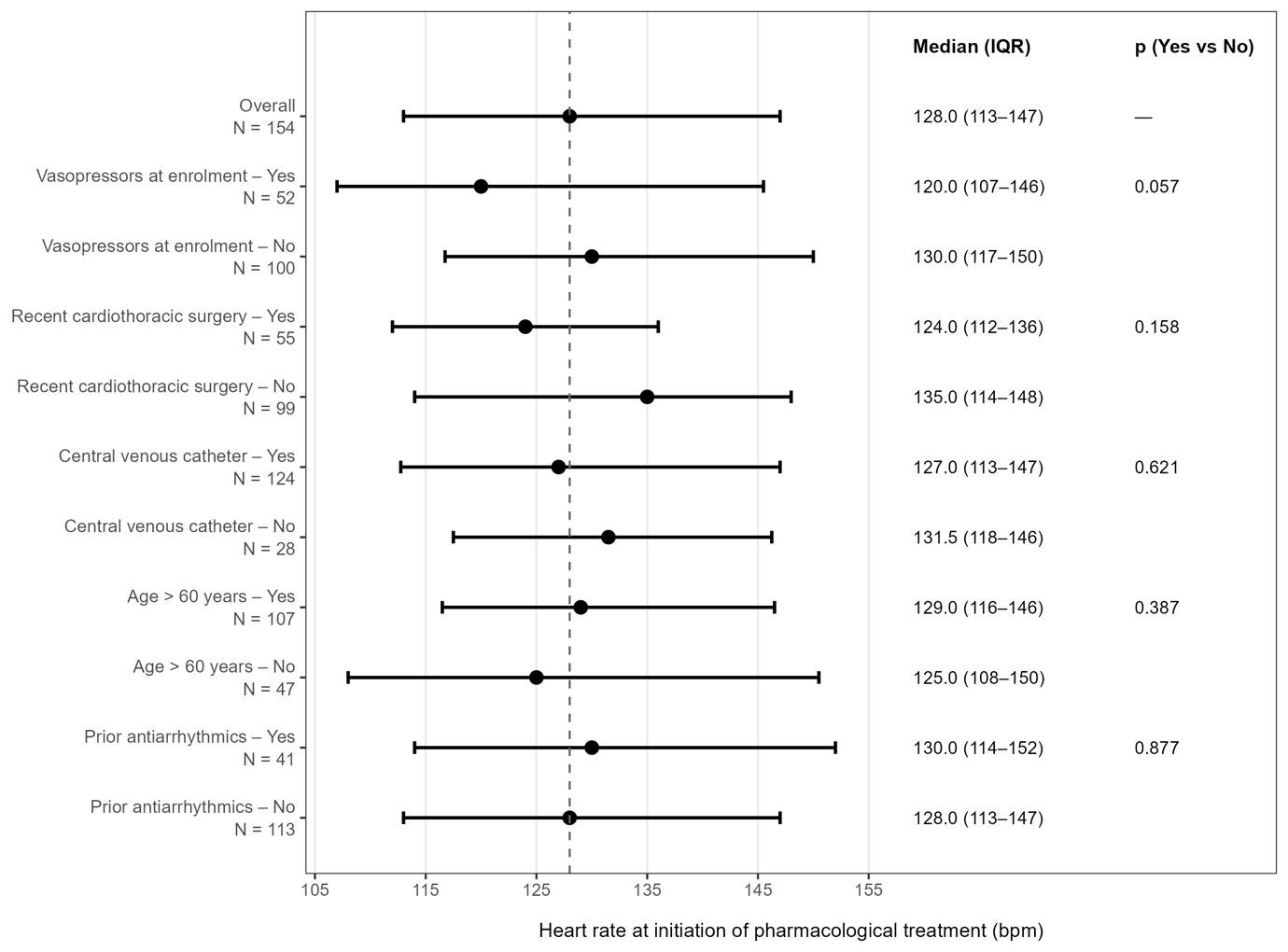


**Figure S2 showing breakdown of first, second and third drugs used during ICU stay**


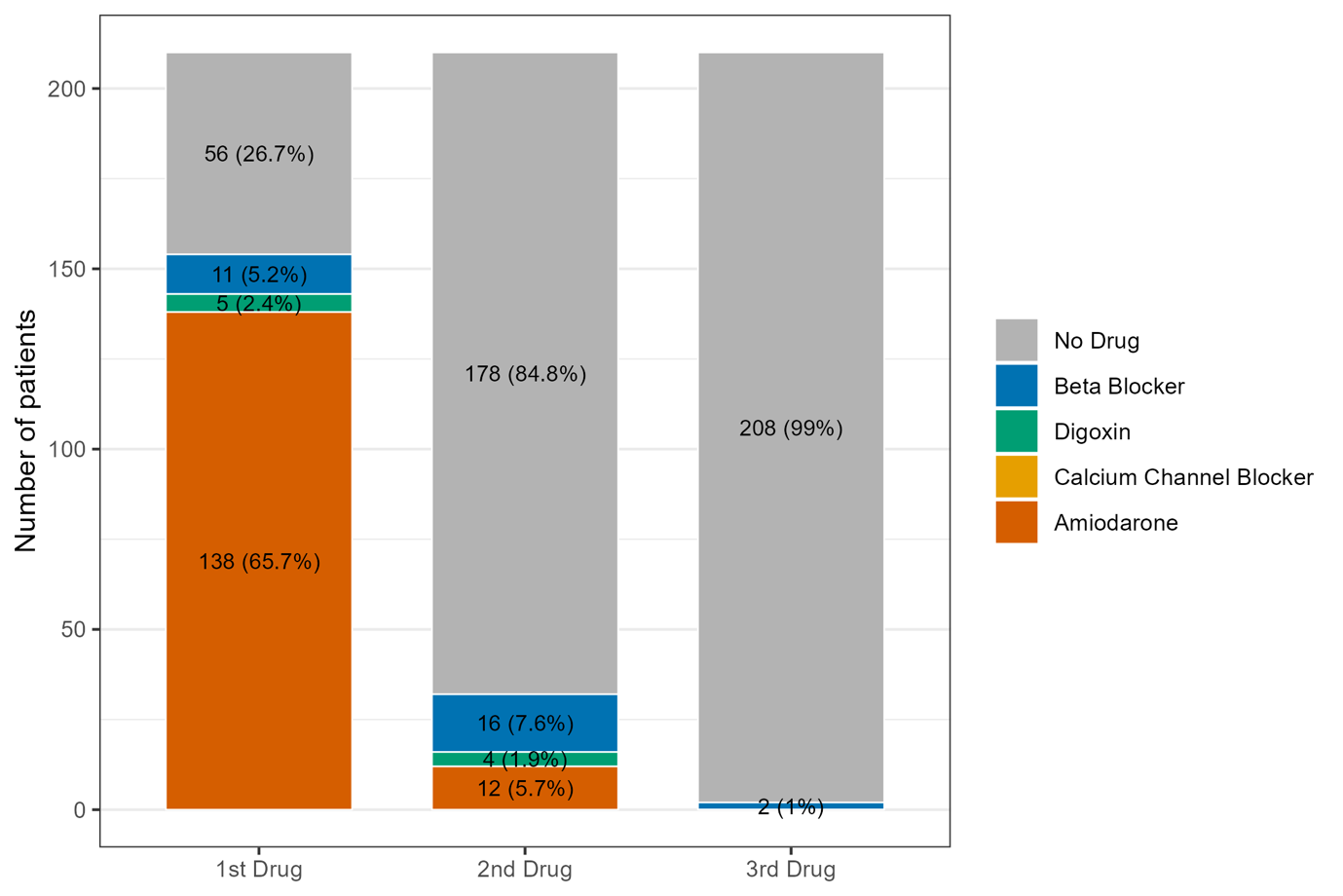


**Figure S3 showing unadjusted cumulative incidence curve for sustained reversion to sinus rhythm stratified by receipt of pharmacological treatment**


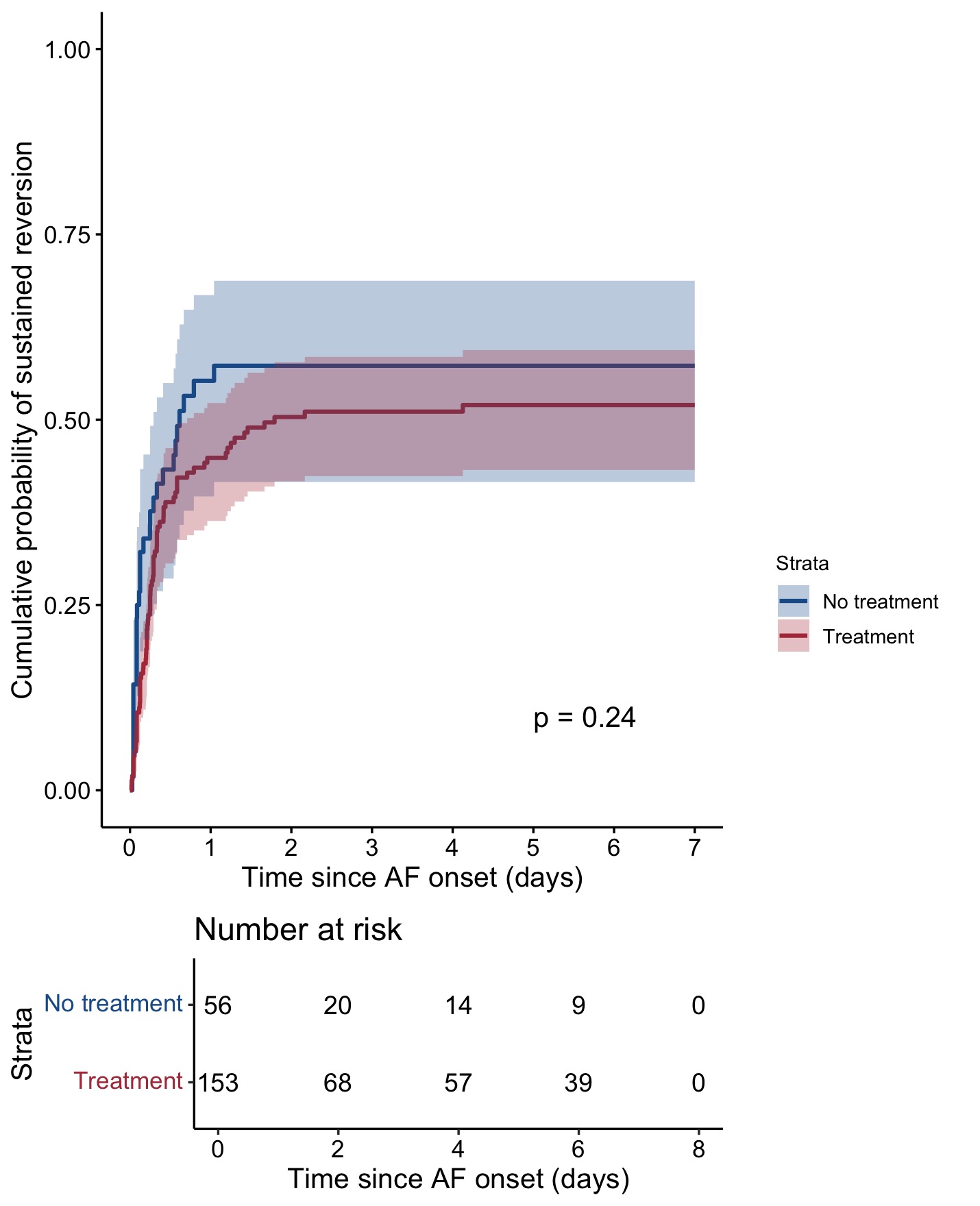


p-value reflects log rank test. Censoring occurred at ICU discharge or 7 days.

**Key Definitions Used**

- ***AF:*** AF is defined as an irregular rhythm (>100bpm) with absence of p waves and irregular RR intervals identified by continuous monitoring or 12-lead ECG lasting at least 30 seconds.
- ***Documented History of AF:*** This includes any documented history of chronic or paroxysmal AF in the medical record prior to ICU admission. This included if AF occurred on the ward immediately prior to ICU admission.
- ***Pharmacological Treatment:*** Pharmacological treatment of NOAF defined as administration of anti-arrhythmic drugs (Digoxin, Beta-blockers, Calcium Channel Blockers, and Amiodarone). This does not include cardioversion in isolation, or the administration of any electrolytes.
- ***Potassium and Magnesium Levels at baseline:*** Use the closest value **preceding** NOAF onset. This can be from a VBG, ABG or formal biochemistry sample.
- ***Maximum HR recorded:*** This is the maximum HR that is documented in the first 24 hours following AF onset.
- ***Co-interventions:*** These must be after the onset of NOAF. Electrolytes are IV or PO. A fluid bolus is any fluid that is given IV that is not a dilutant for a drug or part of a continuous infusion. A change in antimicrobials is either the commencement or change in type or route of antimicrobial charted within 6 hours of AF onset
- ***Heart Rate and MAP at which patient first received pharmacological treatment:*** The recorded value closest to time of pharmacological treatment but **prior** to administration of drug.
- ***Reversion to SR:*** Any documentation that patient was in SR (can be from an ECG, vital sign recording or medical/nursing notes) following onset.
- ***Sustained Reversion to SR:*** Sinus Rhythm must be sustained for 24 hours or discharged from ICU in SR
